# Associations between professional identity and turnover intent in prehospital emergency physicians: The mediating effect of burnout

**DOI:** 10.3389/fpubh.2022.1034925

**Published:** 2022-11-17

**Authors:** Xingmiao Feng, Yu Wang, Pengyu Jia, Yadong Wang, Zhongjun Guan, Kai Meng

**Affiliations:** ^1^School of Public Health, Capital Medical University, Beijing, China; ^2^Beijing Tiantan Hospital, Capital Medical University, Beijing, China

**Keywords:** job burnout, professional identity, mediation effect model, turnover intent, prehospital emergency care

## Abstract

**Context:**

The prehospital emergency system is essential for reducing mortality and disability in emergency patients. However, the high turnover rate of prehospital emergency physicians (PEPs) remains the most prominent problems in the prehospital emergency system. Turnover intent (TI) is predictive of actual turnover behavior; however, previous studies have mainly focused on sociodemographic factors and job characteristics, ignoring many other potential psychological factors, such as professional identity (PI) and job burnout (JB).

**Objectives:**

To measure the level of PI, JB, and TI of PEPs in Beijing, China. We analyze the distribution of TI in different social demography PEPs and then further explore the influence of PI and JB on TI, to provide a reference and suggestions for government departments to reduce the TI of PEPs.

**Methods:**

An online questionnaire was distributed to 552 PEPs in Beijing, and a total of 533 valid questionnaires were included. *T*-test and variance analysis were used to examine the differences in the distribution of TI among different sociodemographic PEPs. Pearson's correlation analysis was used to test the correlation between PI, JB, and TI. The SEM was used to analyze the relationships among PI, JB, and TI.

**Results:**

Univariate analysis showed that age, marital status, education, professional title, work experience, department and hukou were significantly associated with TI. Pearson's correlation analysis showed that PI was negatively associated with JB and TI, and JB was positively associated with TI. Professional treatment identity (PTI, β = −0.24, 95% CI: −0.38~-0.11), professional meaning identity (PMI, β = −0.12, 95% CI: −0.23~0.03), and emotional exhaustion (EE, β = 0.40, 95% CI: 0.28~0.51) seem to have direct impacts on TI. Given the mediating role played by EE, PTI may have an indirect negative effect on TI (β = −0.24, 95% CI: −0.32~0.16).

**Conclusion:**

PI and JB of PEPs in China are closely related to TI, which may have unexpected effects on government departments to stabilize the team of PEPs through a series of control measures. According to the above results, the professional treatment of PEPs needs to be improved, and external learning opportunities should be increased. Legalization of medical rescue workers should also be on the agenda.

## Introduction

### Background

Prehospital emergency refers to emergency first aid provided to patients suffering from various life-threatening emergencies, such as trauma, poisoning, disasters and accidents suffered before arriving at the hospital (1). This work is essential for reducing mortality and disability in emergency patients (2). On the one hand, the study of the Lancet showed that with the change in disease spectrum, stroke and cardiovascular diseases have become the main causes of death in Chinese residents (3). The treatment of stroke and cardiovascular diseases is a race against time, which makes the establishment and improvement of a prehospital emergency care system become very important, and has been widely considered by the society. On the other hand, the global ecological environmental changes, resulting in a variety of public health emergencies and sudden disaster accidents, also show a growing trend, and the prehospital emergency care system has become an important part of the urban security system and public health emergency treatment system (4).

However, high turnover rates and insufficient numbers of prehospital emergency physicians (PEPs) remain the most prominent problems in the prehospital emergency system (4). Studies have shown that PEPs are more likely than other professionals to leave their profession, either by retiring or by pursuing nonemergency clinical practice, management, research, or teaching (5). A study in France showed that the turnover rates of PEPs ranged from 12 to 21.4%, and excessive turnover eventually led to a mass exodus of PEPs (6). One study indicated that the attrition rate for PEPs in the United States within 2 years of completing training was 6.5%, with an estimated average attrition rate of 1.7% PEPs per year (7). Similar results were found in Taiwan, China, where the annual turnover rate of PEPs was 1.83% (8). The prehospital emergency talent team is the foundation of the rapid development of prehospital emergency capabilities, so the establishment and stability of the prehospital emergency team is very important (9). The characteristics of prehospital emergency work (a heavy workload, emergency work, quick transitions, the constant need to perform better and more quickly when dealing with limited resources, uncertainty, a lack of recognition, frustration, and interpersonal conflict) result in PEPs being subjected to tremendous pressure, which can lead to resignation (10). Due to the lack of staff, existing PEPs have to work at full capacity or even overload, which further leads to a high turnover rate, forming a vicious circle (11). Moreover, the grim situation of PEPs shortages led to the prehospital emergency medical services not running a sufficient number of vehicles on duty. Without enough vehicles on duty, it is impossible to set up a tight and reasonable emergency station network covering all areas, resulting in the phenomenon that emergency vehicles arrive at the scene slowly or even have no vehicles to send, which cannot meet the needs of prehospital medical emergency services (4). Therefore, it is urgent to effectively reduce the turnover rate of PEPs and realize the stability of the prehospital emergency physician team.

As early as the early 20th century, some scholars began to study the demission behavior of employees. At present, the concept of demission behavior is divided into a broad sense and a narrow sense. The broad sense refers to “the change of the individual's status as an organization member” (12). In the narrow sense, demission emphasizes that the individual and the organization terminate the labor relationship and leave from the organization, which can be divided into active demission, passive demission and natural demission. Most of the demission behaviors studied by scholars refer to the active demission of employees (13). Turnover intent (TI) refers to the attitude and idea generated by employees to voluntarily leave the organization, which can be used to measure the degree to which employees want to voluntarily leave the organization (14). Mobley believes that to pay attention to the turnover behavior of employees, it is necessary to study their TI first (15). Moreover, TI has appropriate predictability and intervention for actual turnover behavior, and its research value is higher than that of actual turnover behavior (16). Therefore, effectively reducing TI among PEPs is highly important. Previous studies have found that gender, age, education level, work experience, salary and work-family conflicts are all related stress factors for PEPs, which can eventually lead to resignation (17). However, these studies have mainly focused on sociodemographic factors and job characteristics, ignoring many other potential factors. Mobley pointed out that turnover is determined by some basic factors, such as the employee's age and qualification, expectation of the current job, job satisfaction, expectation of other jobs, etc. These factors influence each other and jointly affect individual attitude variables, and attitude variables eventually lead to turnover (18). Therefore, this study included two attitude variables, professional identity (PI) and job burnout (JB), to explore their impact on TI.

PI refers to a person's views concerning a number of factors, such as the social value and goal of the work or task in which he or she is engaged, and this term indicates his or her recognition and understanding of the content, nature and value of this occupation (19). Niemi believes that PI is the self-concept of employees, which refers to the understanding of the nature, content and value of the occupation in the process of long-term engagement in a certain occupation. It is the psychological basis for employees to complete their own work (20). Schein believes that PI is an individual's understanding of his or her occupation and his or her recognition of self-ability development and professional value (21). Foster K et al. noted that PI is crucial to doctors' career development and personal growth (22). Some studies have shown that there is a significant negative correlation between PI and TI, and the higher the degree of PI is, the weaker the TI (23, 24).

Freudenberger was the first to propose JB. He believed that JB is most likely to appear in the helping industry, which is a kind of physical, emotional and behavioral exhaustion caused by long-term work (25). Currently, there are many definitions of JB. Maslach et al. believe that JB is a psychological syndrome that is an emotional expression and response of people to long-term work (26). Most researchers also prefer Maslach's multifaceted definition of JB. Maslach believes that JB includes three dimensions: emotional exhaustion (EE), depersonalization (DP), and low personal achievement (LPA). In the past, JB has been understood as a descriptive disorder; however, it is now recognized in the recently updated International Classification of Diseases, 10th revision, code Z73.0 (27). Multiple studies have shown that JB is closely related to TI, and the higher the degree of JB, the stronger the TI. Failure to identify and solve the underlying causes of JB may lead to health damage and staff shortages of PEPs and may have a negative impact on patient care (27–29).

Therefore, both PI and JB are potential influencing factors affecting the TI of PEPs. However, current studies have mostly focused on the relationships among PI, JB, and job satisfaction (30). Alternatively, studies have explored the factors influencing PI, JB (31) and TI (32), but less attention has been given to the relationships between PI, JB, and TI, especially in the context of prehospital emergency care. A few studies have explored the impact of PI on TI or the impact of JB on TI, but the path analysis of the effects of the two on TI remains to be explored (24, 33). Accordingly, the relationship between PI, JB, and TI was explored by developing a structural equation model (SEM) in this study.

### Goals of this research

The first objective of this study was to understand the current status of the PI, JB, and TI of PEPs in Beijing, China. The second objective was to explore the distribution of TI among different populations of PEPs. The third objective is to explore the relationship between PI, JB, and TI to provide a reference and suggestions for government departments to reduce the TI of PEPs and stabilize the prehospital emergency team in the future.

### Literature review and hypothesis

#### PI and TI

Tajfel (34) introduced the concept of “social identity,” which refers to an individual's knowledge of belonging to a certain social group as well as the emotional and value significance of group membership (35). According to SI theory, employees constantly seek ways to improve their self-esteem and self-image through the groups and organizations to which they belong. If an employee does not feel that the organization helps improve his or her self-perception, then at best the employee will try to change the organization, and at worst the employee will quit (36). In addition, studies have shown that there is a significant negative correlation between PI and TI, and the higher the degree of PI is, the lower the rate of TI (24). Based on this, we propose the following hypothesis:

H1: PI negatively affects TI.

Professional treatment identification (PTI) refers to the identification of physicians on the matching degree between their efforts and gains in the current occupation, including patient identity, work reward, working environment, etc. Adam believes that employees' working attitude depends on effort-reward, longitudinal comparison between themselves and others (37). Adam Smith's economic exchange theory holds that obtaining the use value and economic value from the exchange process is the premise of exchange behavior, and only when the exchange process achieves a win-win situation can the balance be achieved. The essence of exchange is the social exchange of rewards and efforts. Rewards-efforts=result; rewards includes nonmaterial return (awards, honors, status) and material return (income, dividends), and efforts include physical effort and intellectual effort. If both sides of the equation are positive, the exchange relationship is maintained; otherwise, the equilibrium is broken (38). Therefore, if PEPs feel effort-reward imbalance or their income is lower than that of the reference staff, they may produce TI. Based on this, we propose the following hypothesis:

H1a: PTI negatively affects TI.

Professional meaning identification (PMI) is the opposite emotional experience to EE and DP. It is the positive perception of the purpose and value of work. Compared with other occupations, PEPs have irregular working hours, a heavy workload, and great physical and mental pressure, which lead to the excessive burden of family roles and work roles. The original purpose of PEPs is to treat critically ill patients. They hope to obtain more social recognition like that received by medical staff in hospitals. They may be inclined to leave their current job when they feel that the job is not meeting their expectations, that the job is not valuable, and that it is difficult to obtain the corresponding respect (39). Therefore, we propose the following hypothesis:

H1b: PMI negatively affects TI.

Professional ability identification (PAI) refers to physicians' recognition of their knowledge and skills in the medical profession. The career progression of PEPs is the same as that of in-hospital physicians, but the job content is different. Therefore, PEPs are unable to compare with in-hospital physicians in terms of scientific research ability and paper writing ability, and it is more difficult to promote their professional titles, which can easily lead to doubts and negative attitudes in their cognition and evaluation of self-professional value and decrease their enthusiasm for work, thus affecting their on-the-job willingness. Research shows that, in an organization, the higher the degree of self-respect of employees, the greater they think they have important and valuable significance in the organization to which they belong, endowing individuals with inner strength and sense of value and full of confidence and pride in their work to improve job satisfaction and reduce TI (40, 41). Therefore, we propose the following hypothesis:

H1c: PAI negatively affects TI.

#### JB and TI

COR theory posits that employee pressure, performance, burnout and other problems are caused by an imbalance between individual resource input and output. When individuals invest a large amount of original resources, such as time, energy, and social relations, but cannot obtain a comparable replenishment of resources, burnout intent occurs (42). Pines found that the most serious consequence of JB for an organization is the loss of employees (43). Maslach found that among the negative effects of JB, the ultimate and most serious is the increase in employees' TI and the turnover behavior that may result from it. Based on the above views, this study believes that when PEPs are enthusiastic about helping others and have a positive working attitude and constantly invest time, energy, knowledge and technology to provide emergency services but cannot feel their own value in the work, there is a serious imbalance between the effort and reward. The original resource of positive emotions, such as self-efficacy, optimism, positive personality traits resources suffered loss, EE, DP and LPA symptoms of JB, to avoid itself to the “spiral loss,” action must be taken to prevent resources from continuing to be damaged, by giving up current career (44). Therefore, we propose the following hypothesis:

H2: JB positively affects TI.

EE is when a practitioner becomes exhausted by excessive physical and emotional exhaustion and is unable to relax and restore physical and mental exhaustion. It is the most direct response to work stress and major changes at work and is also the core of JB, which can reduce the job satisfaction of practitioners and produce TI (45). Therefore, we propose the following hypothesis:

H2a: EE positively affects TI.

LPA refers to the practitioners' evaluation of their work ability, work performance, and work value. When workers' personal sense of accomplishment is reduced, they will lack confidence in their work and feel that their prospects are slim (45). The need for achievement theory was put forward by American professor David McClelland in the 1950's. McClelland pointed out that in addition to basic physiological needs, there are three kinds of human needs, namely, the need for power, the need for belonging and the need for achievement. Among them, the need for achievement refers to the need to strive to do the best and succeed. People with the need for achievement have a strong desire for success, desire challenges, and want to obtain the satisfaction of success by achieving certain difficult goals. Generally, those who need high achievement are mostly middle class, intellectuals and so on. The subjects of this study are PEPs, most of whom are highly educated. Combined with the previous investigation and research, it is found that their achievement needs are strong. They are willing to do their best work and eager to use what they have learned to help every patient in crisis. Therefore, the high achievement motivation of PEPs is very consistent with the achievement needs theory. When their achievement needs are not met, they will have TI and then seek new jobs. Therefore, we propose the following hypothesis:

H2b: LPA positively affects TI.

#### PI and JB

Furthermore, studies have found that the more an employee PI with his or her current job, the lower the level of JB (46). Positive PI can improve personal satisfaction and reduce JB, thus reducing turnover rates (47). Therefore, we propose the following hypothesis:

H3: PI negatively affects JB.

PTI is an important reason for the TI of PEPs. PEPs have high workload and pressure. When much effort is not satisfied with the reward, it will lead to the formation of negative and indifferent work attitudes of PEPs (48). In addition, in China, especially in Beijing, the capital city, housing loans and children's education expenses and other life pressures are high. If the professional treatment of PEPs cannot meet the needs of life, it will lead to the reduction of personal sense of the accomplishment of PEPs, and then the TI occurs. Therefore, we propose the following hypothesis:

H3a: PTI negatively affects EE.
H3b: PTI negatively affects LPA.

PMI refers to the degree to which physicians identify with the personal meaning and value brought by their current career. According to Miller, when individuals feel respected and proud of their profession, it will have a positive impact on work behavior. This indicates that when the positive value and social evaluation of an individual's career decrease, it will have a negative impact on work attitude and cause a change in career persistence intention. PEPs are under great pressure. If they are unable to perceive the value and significance of their work and obtain a high sense of personal achievement, it is easy to cause excessive physical and psychological consumption, resulting in EE (49). Therefore, we propose the following hypothesis:

H3c: PMI negatively affects EE.
H3d: PMI negatively affects LPA.

The dimension of PAI refers to the judgment of practitioners on their competence and confidence in business execution, which is the “inner maturity” of individuals and the evaluation of their overall sense of competence related to their career. When individuals think that their own ability is not competent for the current job, promotion expectations are difficult to meet or development space is limited, which will reduce their career development expectations and career achievement, leading to the weakening of work passion, and the strengthening of JB. Therefore, we propose the following hypothesis:

H3e: PAI negatively affects TI, and EE plays a mediating role.
H3f: PAI negatively affects TI, and LPA plays a mediating role.

**Figure 1:**
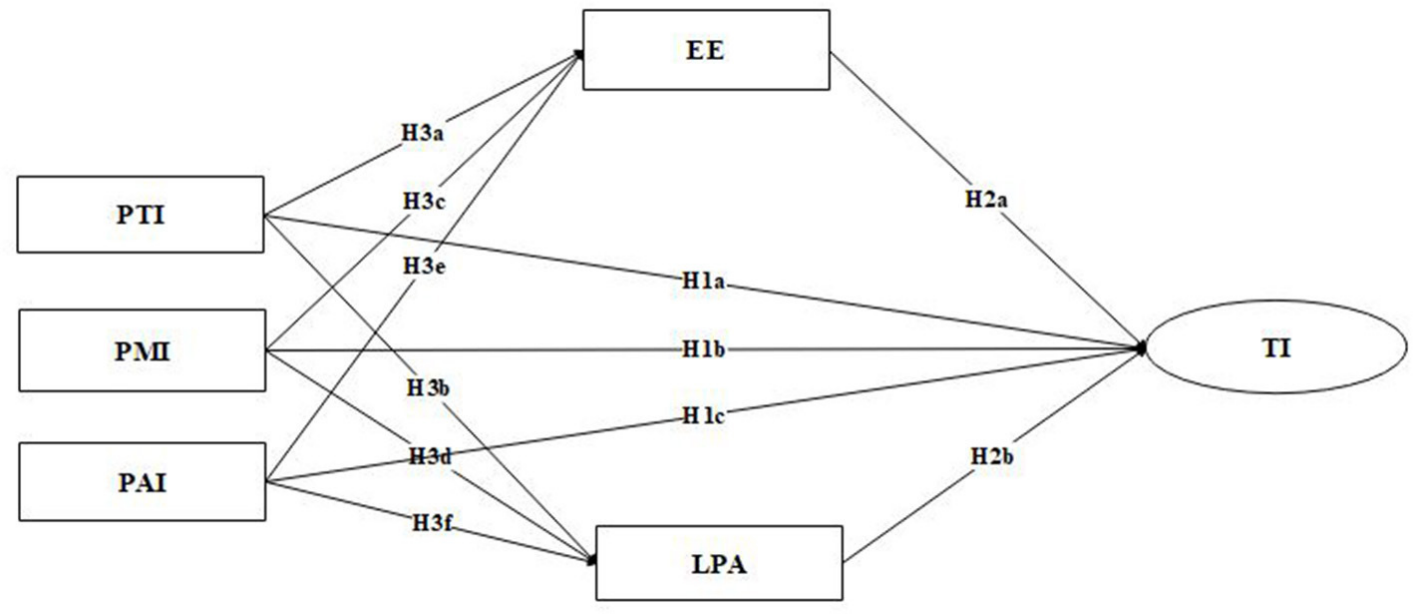
Theoretical model. PTI, professional treatment identity; PMI, professional meaning identity; PAI, professional ability identity; EE, emotional exhaustion; LPA, low personal achievement; TI, turnover intent.

Thus, the hypothesis framework of this study has been formed, as shown in Figure 1.

## Materials and methods

### Setting and participants

The questionnaire was sent to the mobile phones of respondents in the form of a hyperlink in September 2019. After providing informed consent, respondents completed the questionnaire online. According to the structural equation model, the sample size of a survey should be 10–15 times the number of questionnaire items. Thirty-two items were included in this study (9 items for sociodemographic variables, 11 items for PI, 11 items for JB, and 1 item for TI), so the required sample size was 480. Taking into account the possibility of nonresponses, incomplete questionnaires and lost samples, the sample size was increased by 15% for a final minimum acceptable sample size of 552; thus we conducted a cross-sectional survey of 552 PEPs in Beijing by purposive sampling, and a total of 533 valid questionnaires were collected, with a recovery rate of 96.56%.

### Patient and public involvement

Patients or the public were not involved in this study as this research focused solely on PEPs.

### Measurements

#### Sociodemographic data

In this study, the following sociodemographic characteristics were assessed: gender (male, female), age (≤ 30, 31–40, 41–50, ≥51), education (below Undergraduate, undergraduate, postgraduate), marital status (married, unmarried), work experience (≤ 5, 6–10, ≥11), and professional title (primary, medium, senior). In addition, employment type is a kind of employment system that determines the basic personnel relationship between employer and employees in the form of contract, that is, the identity attribute of employee in the unit is determined by signing an employment contract with the unit. In our study, the employment type included permanent and temporary, permanent means once the worker is transferred or assigned to its unit, the worker becomes the unit for life. Temporary means the employee and the employer signed a certain period of labor contract, the employee and the employer have equal labor relations, and both the employee and employer can voluntarily terminate the contract. Hukou refers to the legal document made by the administrative organ in charge of household administration to record and retain the basic information of the household population. In our study, Hukou includes Beijing (Urban), Beijing (Suburban), and Non-Beijing. Department refers to the work unit, including Beijing Emergency Medical Center (hereinafter referred to as 120) and Beijing Red Cross Emergency Rescue Center (hereinafter referred to as 999) in this study.

#### PI scale

This study used the revised PI scale developed by Chen Jing (50). The scale included a total of 13 questions divided into three parts: PTI, PMI, and PAI, with the scale as a whole using a 4-level scoring system. From “strongly disagree” to “strongly agree,” a higher score indicates a higher level of PI. The Cronbach's α values of PTI, PMI, PAI, and the whole scale were 0.859, 0.878, 0.707, and 0.856, respectively, indicating that the reliability of the questionnaire was good. The Kaiser-Meyer-Olkin (KMO) test scores of the PTI, PMI, PAI, and the whole scale were 0.832, 0.722, 0.638, and 0.847, respectively, which indicated that the structural validity of this questionnaire was acceptable.

#### JB scale

In this study, a burnout scale developed by Chaoping Li was used, featuring a total of 15 questions divided into three parts: EE, DP, and LPA, with the scale as a whole using a 7-level scoring system (51). From “never” to “every day,” higher scores indicate higher levels of JB. According to participatory observation and in-depth interviews, Chinese PEPs generally exhibited severe EE and LPA. Therefore, we adopted EE and LPA to measure burnout in PEPs in this study. The Cronbach's α values of EE, LPA, and the whole scale were 0.846, 0.816, and 0.835, respectively, indicating that the reliability of the questionnaire was good. The KMO scores of EE and LPA, overall, were 0.803, 0.841, and 0.8465, respectively, which indicated that the questionnaire had good structural validity.

#### TI

In this questionnaire, given the number of questions and the response effect, we asked a direct question, “Have you considered leaving this work?”, to determine PEPs turnover intent. Studies have shown that the use of one question was a better means of reflecting turnover intent (52). The options used a 4-level scoring system, 1= never, 2= rarely, 3= occasionally, 4= often.

### Data collection and analysis

The Questionnaire Star platform was used to conduct this survey. Participants in the study completed the questionnaire independently and anonymously after encountering the QR code *via* “sweeping” on WeChat.

SPSS 25.0 and Mplus 8.0 were used to analyze the data. Descriptive analysis was used to analyze the sociodemographic characteristics of respondents, and Student's *t* test and analyses of variance were conducted to test for significant differences in PI, JB, and TI score across different subgroups. Pearson's test was used to analyze the correlations among PI, JB, and TI. SEM was used to examine the mediating effect of JB, including a bootstrap test with 5,000 repeated samplings with respect to the significance test of mediating effects, with *p* < 0.05 indicating that the differences were statistically significant. We took TI as the dependent variable; sociodemographic variables as the control variables (dummy variables were set for unordered multicategorical variables); PI (PTI, PMI, PAI) as the independent variable; and JB (EE, LPA) as the mediating variable to establish a structural equation model. The maximum likelihood (ML) method was used to estimate the assumed model parameters, and the model was modified by combining Pearson correlation analysis results and modification indexes (MI). To assess overall model fit, four parameters were used: CFI, TLI, RMSEA, SRMR. If the CFI and TLI values were above 0.90 and the RMSEA and SRMR values were below 0.08, the model fit was acceptable (53).

In addition, since the variables measured in this study were all from the same subjects, it was a self-reported single-source cross-sectional study, so there may be common methodological variation (CMV). In this regard, two measures were adopted to control CMV in this study, namely process control and statistical control. The first is process control; this study used different scale endpoints and formats for the predictor and criterion measures. This reduces method biases caused by commonalities in scale endpoints and anchoring effects (54). A 4-level scoring system was used in the PI scale, a 7-level scoring system in the JB scale and a 4-level scoring system in the TI. At the same time, reverse scoring questions were set in the JB scale to reduce the consistency, deviation and perfunctory answers (55). The second is statistical control. In this study, controlling for the effects of a single unmeasured latent method factor was chosen to examine the degree of CMV in the current data, which was recommended by Widaman (56). This technique models the effect of the method factor on the measures rather than on the latent constructs they represent. The specific operation is to add a first-order factor with all of the measures as indicators to the theoretical model of this study, and this method has been used in many studies (57).

## Results

### Sociodemographic information

A total of 533 people were included in the study, of whom 392 (73.5%) were unmarried; 346 were males (64.9%); 250 (46.9%) were aged 31–40; 311 (58.3%) had a bachelor's degree; 351 (65.9%) had a junior professional title; 324 (60.8%) were temporary staff; 297 (55.7%) had < 5 years of work experience; and 311 (58.3%) were non-Beijing hukou (Table 1).

**Table 1:** Respondent distribution by demographics and work situations.

**Variables**	***N* (%)**
**Gender**	
Male	346 (64.90)
Female	187 (35.10)
**Age (year)**	
≤ 30	190 (35.60)
31–40	250 (46.90)
41–50	83 (15.60)
≥51	10 (1.90)
**Marital status**	
Married	392 (73.50)
Unmarried	141 (26.50)
**Education**	
Below undergraduate	193 (36.20)
Undergraduate	311 (58.30)
Postgraduate	29 (5.40)
**Professional title**	
Primary	351 (65.90)
Medium	164 (30.80)
Senior	18 (3.40)
**Employment type**	
Permanent	209 (39.20)
Temporary	324 (60.80)
**Work experience (year)**	
≤ 5	297 (55.70)
6–10	122 (22.90)
≥11	114 (21.40)
**Department**	
120	364 (68.30)
999	169 (31.70)
**Hukou**	
Beijing (Urban)	75 (14.10)
Beijing (Suburban)	147 (27.60)
Non-Beijing	311 (58.30)

### Controlling for the effects of a single unmeasured latent method factor

As mentioned in the methods section, in this study we controlled for the effects of a single unmeasured latent method factor to examine the extent of CMV in the current data. In this approach, a multifactor measurement model was tested (χ^2^= 828.542, *P* < 0.001, CFI = 0.901, TLI = 0.884, RMSEA = 0.073, df = 82), and a measurement model with an additional method factor was tested (χ^2^= 441.305, *P* < 0.001, CFI = 0.960, TLI = 0.948, RMSEA = 0.049, df = 104). Δχ^2^ < χ^2^(Δdf, 0.95) (Chi-square Critical Value), the results from these analyses indicated that the method factor did not improve the model fit. The results of these tests suggest that CMV is not a pervasive problem in this study.

### Descriptive analysis of PI, JB, and TI

As shown in Table 2, the average PI score was 27.63 ± 5.17, including 9.77 ± 3.12 for PTI, 9.38 ± 1.43 for PMI and 8.48 ± 2.09 for PAI. The average JB score was 49.67 ± 19.60, the average EE score was 27.53 ± 12.54, and the average LPA score was 22.14 ± 11.39. The average TI score was 2.66 ± 1.02.

**Table 2:** Mean values of PI, JB, and TI.

**Variables**	**Mean ±SD**
PI	27.63 ± 5.17
PTI	9.77 ± 3.12
PMI	9.38 ± 1.43
PAI	8.48 ± 2.09
JB	24.84 ± 9.80
EE	27.53 ± 12.54
LPA	22.14 ± 11.39
TI	2.66 ± 1.02

PI, professional identity; PTI, professional treatment identity; PMI, professional meaning identity; PAI, professional ability identity; JB, job burnout; EE, emotional exhaustion; LPA, low personal achievement; TI, turnover intent.

### Single factor analysis of TI

As seen from Table 3, gender had no significant difference in TI, and the highest TI was found between 31 and 40 years old (*P* < 0.001), PEPs who were married had a higher TI than those who were unmarried (*P* < 0.001), PEPs with postgraduate degrees had the highest TI (*P* < 0.001), the TI of PEPs with medium titles was higher than that of PEPs with primary and senior titles (*P* < 0.001), employment type had no significant difference in TI, PEPs with more than 11 years of work experience had the highest TI (*P* < 0.001), the TI of 120 PEPs was higher than that of 999 PEPs, and PEPs with hukou in Beijing (suburban) had the highest TI (*P* < 0.05).

**Table 3:** Single factor analysis of TI.

**Variables**	**TI**	**t/F**	***P***
**Gender**		0.28	0.779
Male	2.67 ± 1.02		
Female	2.64 ± 1.01		
**Age**		19.79	< 0.001
≤ 30	2.24 ± 1.03		
31-40	2.94 ± 0.89		
41-50	2.78 ± 1.01		
≥51	2.40 ± 1.27		
**Marital status**		6.11	< 0.001
Married	2.82 ± 0.97		
Unmarried	2.22 ± 1.00		
**Education**		11.28	< 0.001
Below Undergraduate	2.39 ± 1.01		
Undergraduate	2.79 ± 1.00		
Postgraduate	3.00 ± 0.89		
**Professional title**		10.83	< 0.001
Primary	2.53 ± 1.02		
Medium	2.96 ± 0.95		
Senior	2.39 ± 0.92		
**Employment type**		0.381	0.703
Permanent	2.68 ± 1.03		
Temporary	2.68 ± 1.01		
**Work experience**		7.25	< 0.001
≤ 5	2.51 ± 1.03		
6–10	2.82 ± 0.89		
≥11	2.87 ± 1.04		
**Department**		8.64	< 0.001
120	2.90 ± 0.94		
999	2.13 ± 0.97		
**Hukou**		3.06	0.048
Beijing (Urban)	2.73 ± 1.06		
Beijing (Suburban)	2.81 ± 0.98		
Other	2.57 ± 1.02		

TI, turnover intent.

### Correlation analysis of PI, JB, and TI

Table 4 shows the correlation coefficients among TI, PI and JB in PEPs. Overall, all of these coefficients were statistically significant at the *P* < 0.01 level. Specifically, PI was negatively associated with both JB and TI. Additionally, JB was positively associated with TI, indicating a prerequisite for mediating effect analysis.

**Table 4:** Correlations among TI, PI, and JB.

**Variables**	**M**	**SD**	**EE**	**LPA**	**PTI**	**PMI**	**PAI**	**TI**
**JB**								
EE	27.53	12.54	1					
LPA	22.14	11.39	0.340^**^	1				
**PI**								
PTI	9.77	3.12	−0.601^**^	−0.380^**^	1			
PMI	9.38	1.43	−0.240^**^	−0.342^**^	0.143^**^	1		
PAI	8.48	2.09	−0.517^**^	−0.485^**^	0.523^**^	0.415^**^	1	
TI	2.66	1.02	0.606^**^	0.364^**^	−0.579^**^	−0.167^**^	−0.480^**^	1

^**^p < 0.01; PTI, professional treatment identity; PMI, professional meaning identity; PAI, professional ability identity; EE, emotional exhaustion; LPA, low personal achievement; TI, turnover intent.

### Mediation effect

**Figure 2:**
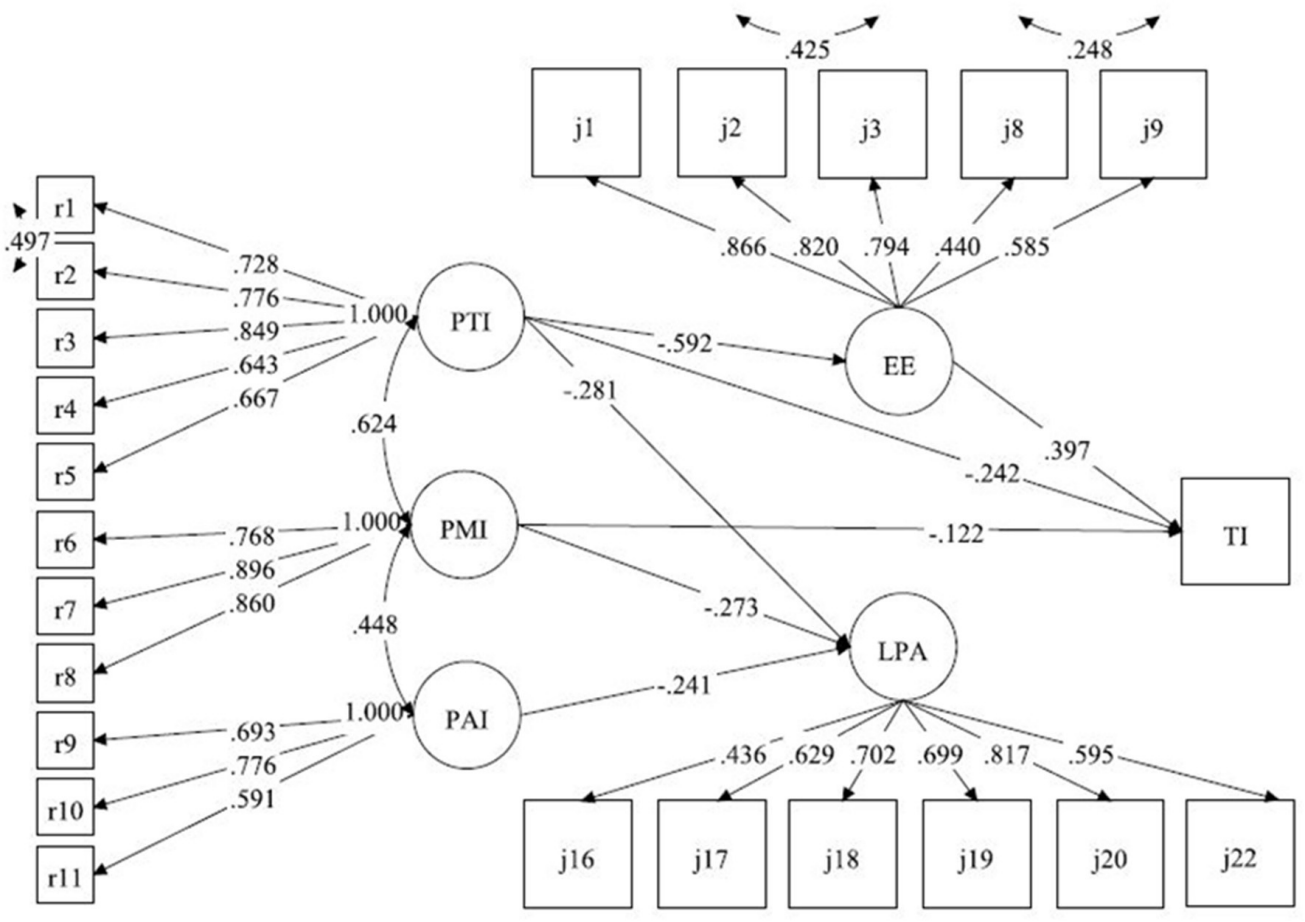
Model paths and standardized coefficients pertaining to the synthesized relationships among PI, JB, and TI. PTI, professional treatment identity; PMI, professional meaning identity; PAI, professional ability identity; EE, emotional exhaustion; LPA, low personal achievement; TI, turnover intent.

The results of the mediation model showed that χ^2^= 655.245, *P* < 0.001, TLI = 0.915, CFI = 0.928, RMSEA = 0.062, SRMR = 0.072, and all fitting indexes were ideal, indicating that the mediation model was acceptable. Figure 2 shows the model path diagram with standardized coefficients, and Table 5 depicts the overall, direct, and indirect effects of the model path. The significance of all effects was tested with a 95% CI estimate.

**Table 5:** Standardized effects of model paths.

**Model paths**	**Estimates**	**95% CI**	***P***
PTI → TI	−0.24	−0.38, −0.11	< 0.001
PMI → TI	−0.12	−0.23, −0.03	0.017
PAI → TI	0.03	−0.07, 0.11	0.497
PTI → EE	−0.59	−0.71, −0.46	< 0.001
PMI → EE	−0.15	−0.28, 0.02	0.053
PAI → EE	−0.08	−0.19, 0.05	0.202
PTI → LPA	−0.28	−0.40, −0.15	< 0.001
PMI → LPA	−0.27	−0.40, −0.12	< 0.001
PAI → LPA	−0.24	−0.38, −0.10	0.001
EE → TI	0.40	0.28, 0.51	< 0.001
LPA → TI	0.07	−0.04, 0.23	0.168
PTI → EE → TI	−0.24	−0.32, −0.16	< 0.001
PTI → LPA → TI	−0.02	−0.05, 0.01	0.208
PMI → EE → TI	−0.06	−0.12, 0.00	0.059
PMI → LPA → TI	−0.02	−0.058, 0.00	0.213
PAI → EE → TI	−0.03	−0.08, 0.02	0.218
PAI → LPA → TI	−0.02	−0.05, 0.00	0.224

PTI, Professional treatment identity; PMI, professional meaning identity; PAI, professional ability identity; EE, emotional exhaustion; LPA, low personal achievement; TI, turnover intent.

When testing the structural model of the sample, we decided to exclude the control variables from the model, since the addition of control variables did not cause substantial changes either in model fitting or in parameter estimates and significance. Additionally, to present the data more intuitively, only statistically significant paths are shown in Figure 2. As seen from the figure, EE (β = 0.40, 95% CI: 0.28~0.51), PTI (β = −0.24, 95% CI: −0.38~-0.11) and PMI (β = −0.12, 95% CI: −0.23~-0.03) had a significant direct impact on TI, while PAI and LPA had no significant direct impact on TI. In terms of indirect effects, PTI (β = −0.24, 95% CI: −0.32~-0.16) mediated the effect of EE on TI.

## Discussion

PEPs are an important aspect of the human resource requirements of prehospital emergency services, and a shortage of healthy, well-trained PEPs can negatively impact the lives and health of patients (28). Therefore, the tasks of effectively reducing TI among PEPs and stabilizing the prehospital emergency team play a very important role with respect to providing high-quality patient care. This study conducted quantitative research concerning the relationships among PI, JB, and TI. PI was divided into three dimensions, PTI, PMI and PAI, and JB was divided into two dimensions, EE and LPA. Then, a mediation model was used to conduct path analysis. Overall, the results of this study showed that 64.7% of PEPs reported a moderate or higher level of TI. Consistent with the literature, our study showed that higher levels of PI among PEPs can reduce TI and that higher JB aggravates TI (5).

### The influence of sociodemographic variables on TI

Univariate analysis showed that the more work experience PEPs had, the higher their TI rates were (*P* < 0.001). McClelland believed that after people's basic needs are met, they pursue high-level needs such as a sense of accomplishment. Therefore, the more work experience PEPs had, the more highly they valued career promotion opportunities. When their current job could not provide promotion opportunities, certain older workers would consider leaving (58). Similarly, PEPs aged 31–40 years (*P* < 0.001) and those who had medium professional titles (*P* < 0.001) pursued career development most avidly and correspondingly exhibited the highest TI rates. In addition, highly educated PEPs usually had relatively high expectations regarding their social status. If their current emergency work could not meet their expectations regarding social status improvement, certain individuals would experience TI (*P* < 0.001). A study by Yi-fang Wu noted that emergency doctors who are married and have children are less likely to leave their jobs, which may be explained by the fact that financial responsibilities related to career change have an excessive impact on family and children, which makes individuals choose to remain in their current jobs (5). In this study, TI among married doctors was significantly higher than that among unmarried doctors (*P* < 0.001). One possible explanation for this result is that married PEPs put their families first, but emergency work often leads to work-family conflict. Multiple studies have shown that work-family conflict is significantly associated with TI and that employees who have greater control over their working hours and more flexibility are less likely to quit (59). Simultaneously, there are essential differences between TI and actual turnover behavior; high TI does not necessarily entail that an individual will leave his or her current career.

### The direct influence of PI on TI

Many scholars have confirmed that PI has a significant negative impact on TI. The lower the level of PI is, the higher TI rates are (60). In this study, PI was divided into three dimensions: PTI, PMI and PAI. There was no significant effect of PAI on TI (β = 0.03, 95% CI: −0.07~0.11), indicating that PEPs in Beijing recognized their ability to engage in prehospital emergency work and believed that they had sufficient professional knowledge and skills to meet the needs of prehospital emergency work. PTI had a significant negative impact on TI (β= −0.24, 95% CI: −0.38~−0.11), and PTI had the lowest score among the three dimensions of PI, indicating that current PEPs were not satisfied with their salaries. Based on their excess contributions to the work of the organization, employees were eager to obtain a reward appropriate to their efforts. When their expectations are not met, their satisfaction with salary, the professional promotion system and the incentive system are reduced, which can lead to resignation, supporting hypothesis H1a (61). PMI had a significant negative influence on TI (β = −0.12, 95% CI: −0.23~−0.03), supporting hypothesis H1b. The preliminary study found that most PEPs in Beijing were medical college graduates with medical qualifications, and they hoped to obtain more social recognition and better career development opportunities. However, the content of their work mostly consisted of simple, routine medical activities, and they found it difficult to sense the value of their work, which can result in resignation.

### The negative influence of PI on JB

The results showed that PMI (β = −0.27, 95% CI: −0.40~−0.12) had a direct negative effect on LPA, supporting hypothesis H3d. LPA refers to low productivity and low self-esteem. Studies have pointed out that although LPA is often excluded from medical burnout surveys, LPA among physicians is prevalent and may worsen (62, 63). In this study, PEPs in both 120 and 999 units showed moderate or higher levels of LPA. A low level of social recognition and a lack of cooperation among relevant social personnel and organizations leads to a decline in the PMI of PEPs. At the same time, as the general population ages, the number of empty nesters and old persons who live alone is increasing, so demand for patient transport is also increasing. However, 120 is the only institution in Beijing that employs (with its own funds) approximately 50 stretcher-bearers to carry out first aid tasks and provide carrying services, while 999 employs no stretcher-bearers (4). Therefore, the task of carrying patients is often the responsibility of PEPs, even though this task is inconsistent with their original intention in choosing emergency work. They originally took up their job of providing prehospital emergency work due to the achievement satisfaction that they could experience through saving the dying and healing the wounded. When they are required to engage in simple transportation and lifting work, it is difficult for these PEPs to meet their personal achievement needs.

The results showed that PTI (β = −0.28, 95% CI: −0.40~−0.15) had a direct negative effect on LPA, which was consistent with the views of previous scholars, supporting hypothesis H3a. Siegrist believes that employees who receive less than they give can experience work-related stress, resulting in a sense of effort-reward imbalance. The effort dimension includes the time and energy spent by employees in their work, and the reward dimension includes aspects such as career prospects, job security and income (64). When PEPs feel that their excess efforts have not been reasonably rewarded, they will experience a sense of loss, and their needs for personal achievement cannot be satisfied effectively (60).

The results showed that PAI (β = −0.24, 95% CI: −0.38~−0.10) had a direct negative effect on LPA, supporting hypothesis H3f. Herzberg's dual-factor theory proposes that incentives such as income security can only eliminate dissatisfaction at work, while incentives such as job development can improve work enthusiasm (65). However, the nature of prehospital emergency care requires that PEPs can only observe the development of and change in patients' conditions over a short period of time, and they cannot track the long-term outcomes of disease treatment or the intervention effects of drugs and other treatment methods. Therefore, it is difficult for the knowledge level and technical skills of PEPs to keep pace with developments in modern medicine due to their long-term involvement in prehospital emergency work (66). Especially under the current professional title promotion system in China, the professional title promotion of PEPs is difficult, the development space is limited, and the personal value is not reflected, resulting in LPA.

### The mediating role of EE on TI

EE refers to feelings of mental or cognitive exhaustion caused by work and is characterized by feelings of fatigue and low emotional energy (67). The results of this study showed that PTI could indirectly affect TI through EE (β = −0.24, 95% CI: −0.32~−0.16). According to COR theory, the mobilization of individual energy and other resources to meet long-term needs leads to a phenomenon of “spiral loss,” which affects resource reserves. When individuals suffer from unmet long-term needs, their energy resources deplete over time, eventually leading to EE (42). The meta-analysis of Lee and Ashforth further confirmed that needs were positively correlated with EE, while resources, such as various types of support and intrinsic motivation at work, were negatively correlated with EE (68). In addition, according to goal-setting theory, resources increase external motivation at work by promoting the achievement of goals. Therefore, when there are insufficient resources to meet the long-term demands of the work, this situation can hinder the achievement of the work goals. When employees are frustrated by difficulties with respect to achieving their work goals, leaving their current job becomes an important self-protection mechanism (69). Self-determination theory also suggests that a lack of resources associated with human needs may encourage disengagement from an intrinsically unsatisfactory career (70). In other words, when faced with great work pressure, if professional treatment fails to provide effective incentives, it is likely that employees will decrease their external motivation, produce EE, and eventually lead to their departure from their current position.

### Implications for PEPs management

Most studies of burnout and turnover in emergency care contexts have focused on solutions that require individual physician action (e.g., meditation, yoga, or physical exercise) (71). While individualized interventions, such as mindfulness and mental health programs, can be palliative, this individual-focused approach can inadvertently shift the blame onto the victim. Moreover, changes at the organizational level can simultaneously affect more prehospital responders and have a greater positive impact. Therefore, it is necessary to find meaningful and lasting solutions at the organizational and systematic levels (28). Through this study exploring the three dimensions of PI (PTI, PMI and PAI), JB in two dimensions (EE and LPA) and the effects of the relationships among PI, JB, and TI, the following conclusions are drawn. First, PTI has a direct negative effect on TI and LPA, and can indirectly affect TI through EE. It is suggested that government departments should improve the work treatment and establish a special subsidy policy for PEPs to improve the PTI and reduce the TI of PEPs and stabilize the prehospital emergency medical team. Second, PMI has a direct negative impact on LPA. In this regard, we suggest that the Chinese government should actively explore the establishment of a development model for medical rescue workers and issue relevant laws and regulations to ensure the legalization of medical rescue workers with certificates. In this way, the medical rescue workers could undertake the transport and lifting work, and the PEPs could undertake the wounded rescue work to improve the PMI of the PEPs and achieve the improvement of their personal sense of achievement of the PEPs. Finally, PAI has a direct negative impact on LPA. In view of this result, we suggest that the relevant prehospital emergency care institutions should provide PEPs with standardized training, provide them with external learning opportunities, and improve their professional skills and abilities in various aspects to enhance both the PAI of PEPs and their personal sense of accomplishment.

## Limitations

The study faces several limitations. First, the design of cross-sectional studies limits the possibility of establishing causal relationships among study variables. In the future, researchers can conduct longitudinal in-depth studies on PEPs to further verify the results of this study and provide a reference for stabilizing the prehospital emergency team.

Second, the scope of this survey was limited to Beijing. Beijing is the capital of China, and its economic level and policy sensitivity are higher than those of other regions in China. Therefore, in the case of limited time and energy, this study selected Beijing as the research site to understand the core situation of PEPs in China. However, at present, China's prehospital emergency care model is based on the structural attribute classification of the executive body emergency care center, which is mainly divided into four types, namely command type, dependent type, independent type and comprehensive type (72). In addition, the development level of prehospital emergency work is not balanced across the country, and a variety of prehospital emergency care models coexist, and even a variety of systems exist in some provinces and cities (with different ownership of institutions). In the future, it is worth further exploring whether the different modes of prehospital emergency care among different regions will affect the TI of PEPs. Therefore, the results of this study need to be replicated with a more representative cross-sectional sample in other areas in the future.

Third, in this research we controlled for the effects of a single unmeasured latent method factor control to test the CMV. Although controlling for the effects of a single unmeasured latent method factor controls for any systematic variance among the items that is independent of the covariance due to the constructs of interest, it does not permit the researcher to identify the specific cause of the method bias. Indeed, the factor may reflect not only different types of CMV but also variance due to relationships between the constructs other than the one hypothesized. Another disadvantage is that this technique assumes that the method factor does not interact with trait factors. Therefore, although this technique can control CMV, it cannot completely eliminate the influence of CMV.

## Conclusion

In this study we found that PTI and PMI have a direct negative effect on TI, and EE has a direct positive influence on TI. PTI, PMI and PAI have a direct negative impact on LPA, and PTI indirectly affects TI through EE. It is suggested that PI and JB may be the potential ways to reduce the TI of PEPs. In view of the above results, efforts should be made by the government departments to develop strategies to decrease the TI of PEPs and stabilize the prehospital emergency team, such as improving the working treatment of PEPs, providing them with external learning opportunities, and actively exploring and establishing the development model of medical rescue workers.

## Data availability statement

The datasets presented in this article are not readily available because confidentiality of pre-hospital emergency worker information. Requests to access the datasets should be directed to mengkai@ccmu.edu.cn.

## Ethics statement

The study was approved by the Ethical Review Committee of the Capital Medical University (Z2019SY057). Participation in the survey was completely voluntary and written consent was obtained from participants.

## Author contributions

XF took charge of the formal analysis and wrote the original draft. YuW took care of the methodology part. PJ was responsibility for the data collection and curation. YaW was responsibility for the data collection and field research. ZG was responsible for the project administration and resources. KM designed the project and took responsibility for the paper as a whole. All authors contributed substantially to article revisions. All authors contributed to the article and approved the submitted version.

## Funding

This study was supported by Beijing Municipal Committee of the Chinese People's Political Consultative Conference in 2019.

## Conflict of interest

The authors declare that the research was conducted in the absence of any commercial or financial relationships that could be construed as a potential conflict of interest.

## Publisher's note

All claims expressed in this article are solely those of the authors and do not necessarily represent those of their affiliated organizations, or those of the publisher, the editors and the reviewers. Any product that may be evaluated in this article, or claim that may be made by its manufacturer, is not guaranteed or endorsed by the publisher.
